# *Kcnk3, Ggta1,* and *Gpr84* are involved in hyperbaric oxygenation preconditioning protection on cerebral ischemia–reperfusion injury

**DOI:** 10.1007/s00221-021-06220-7

**Published:** 2021-09-30

**Authors:** Chunhui Yang, Minjun Ding, Guiqiang Shao, Shengjie Jia, Xue Yin, Yuhui Cui, Zetian Wang, Chunrong Wu

**Affiliations:** 1 Department of Trauma-Emergency and Critical Care Medicine Center (TECCMC), The Fifth People Hospital of Shanghai Fudan University, Shanghai, 200240 China; 2 Department of Hyperbaric Oxygen Chamber, Wujing Hospital, Minhang District, Shanghai, 200241 China

**Keywords:** Middle cerebral artery occlusion/reperfusion, Cerebral ischemia and reperfusion injury, *Ggta1*, *Gpr84*, *Kcnk3*, Hyperbaric oxygenation preconditioning

## Abstract

The present study aimed to explore the potential mechanism of the effect of hyperbaric oxygenation (HBO) preconditioning on cerebral ischemia and reperfusion injury (CIRI). GSE23160 dataset was used to identify differentially expressed genes (DEGs) from striatum between the middle cerebral artery occlusion (MCAO)/reperfusion and sham rats. The gene clusters with continuous increase and decrease were identified by soft clustering analysis in Mfuzz, and functional enrichment analysis of these genes was performed using clusterProfiler package. The intersection set of the genes with significantly altered expression at post-reperfusion 2, 8, and 24 h were screened in comparison to 0 h (sham group), and the expression of these genes was detected in the MCAO/reperfusion model and HBO preconditioning groups by real-time PCR (RT-PCR) and western blotting. A total of 41 upregulated DEGs, and 7 downregulated DEGs were detected, among which the expression of *Gpr84* and *Ggta1* was significantly upregulated at each reperfusion phase as compared to the sham group, while the expression of *Kcnk3* was significantly downregulated except in the postreperfusion 8 h in the striatum group. RT-PCR and western blotting analyses showed that the expression of *Ggta1*, *Gpr84*, and *Kcnk3* genes between the MCAO/reperfusion and sham rats were consistent with the bioinformatics analysis. In addition, the HBO preconditioning reduced the expression of *Ggta1* and *Gpr84* and increased the expression of *Kcnk3* in MCAO/reperfusion rats. *Kcnk3*, *Ggta1*, and *Gpr84* may play a major role in HBO-mediated protection of the brain against CIRI.

## Introduction

Stroke is one of the three major diseases that threaten human health, and the second-leading cause of death globally (Gorelick 2019). A total of 80.1 million stroke cases and 5.5 million deaths were recorded globally in 2016. The age-related incidences of stroke are highest in east Asia, especially China, followed by eastern Europe, ranging from 200 to 354/100,000 person-years (Johnson et al. 2019). In addition to the high fatality rate, stroke also causes a high disability rate, which might bring a great health burden to society (Lancet 2013). Accumulating evidence has indicated that several risk factors, such as hyperlipidemia, hypertension, and smoking, are responsible for the development of stroke (Kumar et al. 2016; Lu et al. 2019). Due to the increased mortality of stroke, the pathogenic mechanisms induced by risk factors need to elucidated urgently for the prevention and treatment of stroke.

Cerebral ischemia and reperfusion (I/R) injury (CIRI) following stroke aggravates brain damage when blood supply is restored (Venkat et al. 2017). Reportedly, inflammatory responses, production of free oxygen free radical, and disruption of brain-blood barrier (BBB) (Lin et al. 2016; Sun et al. 2018) are the primary events for stroke-induced CIRI. Since CIRI is one of the major causes of lethality, the prevention and alleviation of CIRI constitute the key processes for stroke research. Several therapies, such as electroacupuncture (Huang et al. 2019) and hyperbaric oxygenation (HBO) preconditioning (Hentia et al. 2018) are currently applied to alleviate CIRI. HBO treatment is an efficient method to ameliorate CIRI via reducing the infarct volume (Lou et al. 2004). Moreover, HBO therapy reduces BBB damage, improves endothelial function and rheology, and decreases local inflammation and edema after CIRI (Hentia et al. 2018; Sun et al. 2018). However, the exact molecular mechanisms of HBO treatment that affect CIRI are only partially known.

The whole transcriptome sequencing is a robust technique for characterizing and quantifying the transcriptome in different stages using bioinformatics analysis (Yang and Kim 2015). In the present study, the transcriptome expression data of GSE23160 dataset that comprised by 24 brain tissue samples from striatum and cortex of post-I/R injured areas at 2, 8, and 24 h post-reperfusion were downloaded to identify the CIRI-related differentially expressed genes (DEGs) in the rat middle cerebral artery occlusion (MCAO)/reperfusion model as compared to the sham group (Chen et al. 2011) and analyzed their functions. Then, we evaluated the effect of HBO preconditioning on several key DEGs in the rat MCAO/reperfusion model of ischemia by real-time PCR (RT-PCR) and western blotting. The present study aimed to explore the potential treatment targets of CIRI and investigate the molecular mechanisms of HBO preconditioning to ameliorate the cardiac function after CIRI.

## Methods

### Microarray data

The GSE23160 dataset was downloaded from the Gene Expression Omnibus (GEO, http://www.ncbi.nlm.nih.gov/geo/) and produced on the platform of the Illumina MouseRef-8 v2.0 expression beadchip (GPL6885). The dataset consisted of 24 brain tissue samples from right striatum and cortex (corresponded to infarct area) of post-I/R injured areas, which were extracted at 2, 8, and 24 h post-reperfusion (*n* = 4), respectively, and 4 brain tissue samples each from the striatum and cortex in the sham group.

### Data preprocessing

The original data were read and preprocessed by using Linear Models for Microarray Data (limma, version 3.32.2, https://bioconductor.org/packages/) package in R software (version 3.4.0) (Smyth 2005), in which, the RMA (robust multi-array average) method was applied to convert the probe level data to expression value with background-adjusted, quantile normalization, and log2 transformed values of perfect-match intensities (Irizarry et al. 2003). Then, the probes were annotated. If different probes mapped to the same gene symbol, the mean expression value of those probes was considered as the expression value of the specific gene.

### Identification of DEGs and hierarchical cluster analysis

After preprocessing the data, limma package was utilized for the analysis of the differential expression of genes between cortex and striatum post -2, -8, and -24 h I/R groups as compared to the corresponding cortex and striatum of sham groups, respectively. The cut-off criterion for screening DEGs was defined as *P*-value < 0.05 and |log_2_ fold change (FC)|> 0.585. Then, the common DEGs identified between 2 h *vs*. sham, 8 h *vs*. sham, and 24 h *vs*. sham from the cortex and striatum tissue samples were obtained, respectively. In addition, the cluster heatmap was drawn using pheatmap package in R, followed by a complete-linkage clustering method with Pearson’s correlation distance.

### Soft clustering analysis

Soft clustering is more accurate than hard clustering with more robustness to noise and less information loss and can assign a gene to several clusters using the fuzzy c-means algorithm with time-course data on the gene expression (Futschik and Carlisle 2005; Kumar 2007). Mfuzz is one of the software packages for soft clustering, in which the membership values of genes in a cluster is generated for reflecting the strength of a gene's association with a cluster (Kumar 2007). Membership value can reveal the similarity of gene expression vectors to each other. To analyze the expression trend of common DEGs with I/R time changes in both cortex and striatum groups, the Mfuzz package (version 2.36.0, https://bioconductor.org/packages/release/bioc/html/Mfuzz.html) in R was used to perform noise-robust soft clustering analysis that could be used for visualizing the time-dependent expression patterns of genes. According to the changes in the gene expression with time, soft clustering could assign genes to several cluster modules, such as continuous increase, continuous decrease, increase first then decrease, and decrease first then increase. The analysis parameters were set as minimum standard deviation = 0.3; score (membership values) = 0.5, and c Optimal = 8. The modules with a continuous increase or continuous decrease were selected for subsequent analysis.

### Functional enrichment analysis

Gene ontology (GO, http://www.geneontology.org) analysis has been widely used to annotate the gene function in biological processes, molecular functions, and cellular component categories (Hulsegge et al. 2009). Kyoto Encyclopedia of Genes and Genomes (KEGG, http://www.genome.jp/kegg) is a database containing the information of genes related to metabolic and regulatory pathways (Kanehisa et al. 2017). In the present study, the clusterProfiler package (version 3.4.4, https://bioconductor.org/packages/release/ bioc/html/clusterProfiler.html) in R was used to conduct the functional analysis for continuous upregulated and downregulated DEGs in both cortex and striatum groups at different I/R times, respectively. The P-value of enriched pathways was calculated by a hypergeometric distribution test. Subsequently, the *P*-value was revised using BH (Benjamini and Hochberg) method, and the adjusted *P*-value < 0.05 was served as the cut-off criterion for selecting significant enrichment pathways.

### Identification of key DEGs in both cortex and striatum groups

Based on soft clustering, the gene clusters were acquired. Then, overlapped DEGs with continuous upregulation and downregulation in both groups were identified by Venn diagram using Venny 2.1 online tool. Next, we used clusterProfiler package with the same threshold value, as mentioned above, to conduct the GO and KEGG analyses. Finally, several overlapped DEGs with significantly different results were screened further and changed to homologous genes in rats. These overlapped DEGs were identified as key I/R-related genes for the subsequent experimental validations followed in this study.

### Animals

A total of 24 male Sprague–Dawley rats (200–260 g, 9–11-weeks-old) were purchased from Shanghai SLAC Laboratory Animal Co., Ltd. The rats were allowed free access to food and water under controlled conditions (room temperature 24 ± 2 °C, humidity 50 ± 5%, and lighting with 12-h light/dark cycle). After a week of adaptive feeding, the animals were randomly and equally divided into three groups: MCAO/reperfusion, sham operation, and HBO preconditioning + MCAO/reperfusion. All procedures were approved by the Animal Ethics Committee and performed in accordance with the committee guidelines.

### Focal cerebral ischemia–reperfusion model and HBO treatment

MCAO/reperfusion model was used to simulate the I/R injury in rats by referencing reforming Longa method (Longa et al. 1989). Briefly, rats were anesthetized intraperitoneally using 3% pentobarbital (50 mg/kg, Shanghai Rongbai Biological Technology Co., Ltd, China). The left common carotid artery (CCA), external carotid artery (ECA), and internal carotid artery (ICA) were dissociated following a midline neck incision. Then, the proximal part of CCA was clamped, and the ECA was ligatured. A nylon suture (Beijing Cinontech Co., Ltd; 2432A3, Beijing, China) was inserted in the carotid bifurcation of CCA, avoiding the pterygopalatine artery, to enter the internal carotid artery, and it was about 17–20 mm distal to the middle cerebral artery. After a 2-h ischemic period, reperfusion was performed by withdrawing the suture to restore the blood supply, and rats were euthanized for 24 h post-reperfusion. The same procedures were conducted in the sham group, except suture insertion was performed on rats.

In the HBO preconditioning + MCAO/reperfusion group, animals were firstly treated with HBO in the pressurized oxygen chamber. After flushing pure oxygen until an oxygen pressure of 0.1 MPa was achieved, the rats were subjected to 2.0 atmospheres absolute (ATA) (0.2 MPa) under pure oxygen for 1 h. After 15 min of constant decompression, the pressure decreased to normal, and the rats were discharged from the chamber. The rats were pretreated with this method once a day for 7 consecutive days. Next, after 3 h of the last HBO preconditioning, the rats were utilized to establish MCAO/reperfusion models.

### RT-PCR analysis

Approximately, 50–100 mg brain (right motor cortex region) samples were obtained from each rat and homogenized. Total RNA was extracted using RNAiso Plus kit (Cat. 9109, TaKaRa, Dalian, China), and reverse transcribed into complementary DNA (cDNA). Then, the quantitative real-time PCR reactions were conducted using Power SYBR Green PCR Master (4,367,659, Applied Biosystems Inc., CA, USA). The reaction conditions were as follows: 95 °C for 3 min, 95 °C for 10 s, and 40 cycles at 60 °C for 30 s. The primers of the detected genes are listed in Table 1; *GAPDH* was used as the reference gene. The relative expression of the genes was calculated by the 2^−ΔΔCt^ method. All reactions were repeated three times.

**Table 1:** The primer sequence for each validated gene

Primer name	Primer sequence (5′-3′)
GAPDH-rF	AGACAGCCGCATCTTCTTGT
GAPDH-rR	CTTGCCGTGGGTAGAGTCAT
Ggta1p-rF	ACCGATTCTGCTGAAGACCT
Ggta1p-rR	CAAACAGCAGAGCAACCGAG
Gpr84-rF	TTCGGACTCCTCCTCTTTACT
Gpr84-rR	ACAACTGGCACCAAGACATAA
Kcnk3-rF	GCTCCTTCTACTTCGCCATCA
Kcnk3-rR	TGACTAGTGTGAGCGGGATG

### Western blotting

The protein levels of key genes in brain tissues samples were analyzed by western blotting. The tissue protein extracts were subjected to SDS-PAGE and electrophoresed proteins were then transferred onto PVDF membranes. Antibodies for Gpr84 and Kcnk3 were added to the membranes and the membranes were incubated overnight at 4 °C. The membranes were then washed 3 times, secondary antibodies (anti-rabbit: 1:10,000 and anti-mouse: 1: 5000; Jackson, American) were added, and samples were incubated for 2 h at room temperature. A western blot imager was used to observe the protein bands after the blots were further washed and developed using an ECL substrate (Beyotime, Nanjing, China).

### Statistical analysis

The expression values were presented as means ± standard error of mean (SEM). The statistical significance among different groups was analyzed by one-way analysis of variance (ANOVA) with Newman–Keuls multiple comparison posttest, and *P* < 0.05 was considered statistically significant. All analyses were conducted using SPSS 22.0 software (IBM Corporation, Armonk, NY, USA), and GraphPad Prism 5 (GraphPad Software, Inc., La Jolla, CA, USA) was utilized to construct graphs.

## Results

### Identification of DEGs and hierarchical cluster analysis

**Fig. 1:**
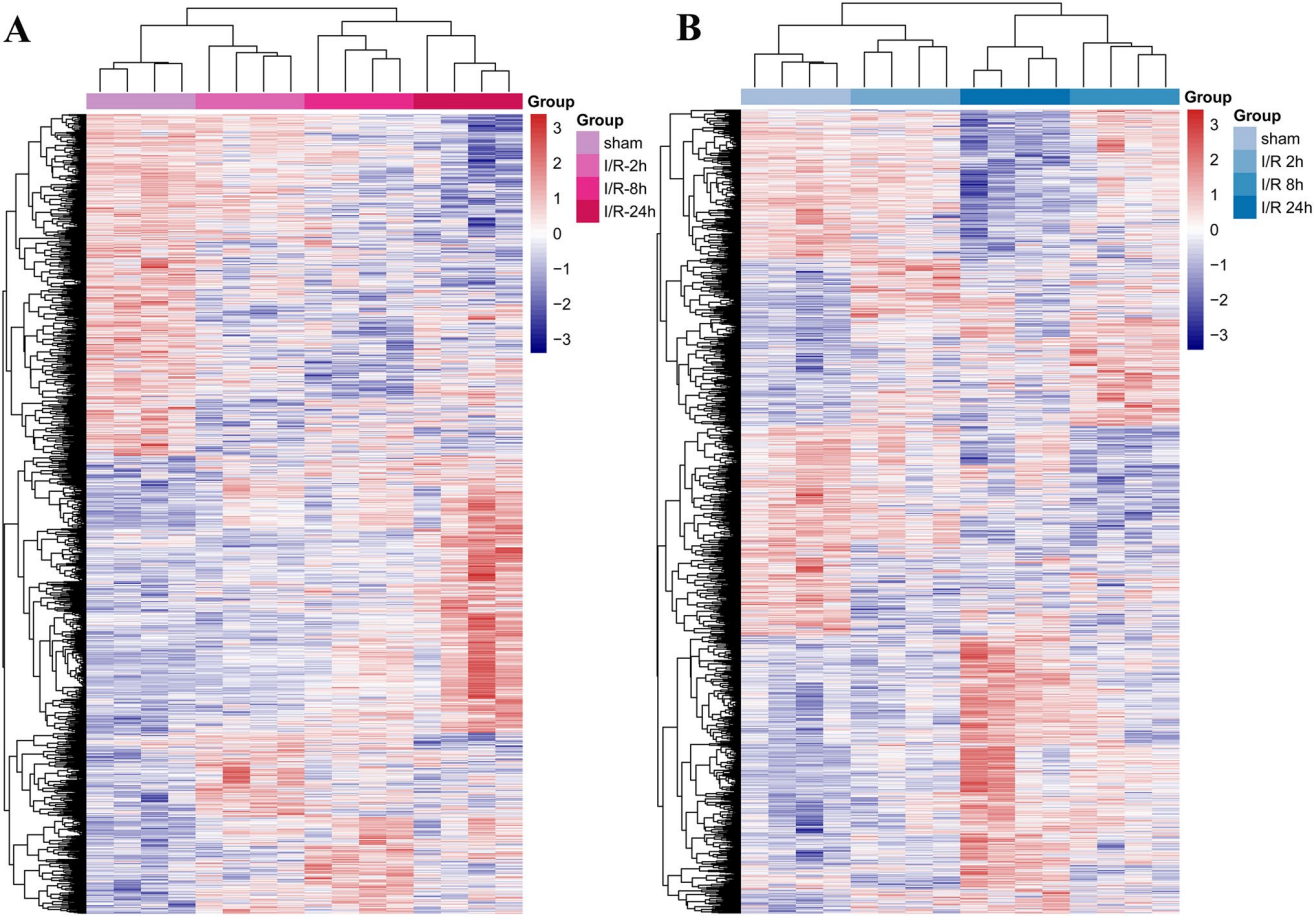
Hierarchical cluster map of DEGs identified in the cortex (**A**) and striatum (**B**) groups. The horizontal axis denotes samples, and the right vertical axis shows clusters of DEGs. The smoothly changed color from blue to red encodes expression values from low to high

The GSE23160 dataset consists of 26,534 probes corresponding to 18,120 genes. The DEGs identified in the cortex and striatum groups at different reperfusion times are listed in Table 2. A total of 673, 910, and 1414 DEGs were identified in the cortex, and 590, 1062, and 1596 DEGs were identified in the striatum at 2, 8, and 24 h post-reperfusion, respectively. With the extension of reperfusion time, the number of DEGs increased gradually (Table 2). Compared to the cortex-sham group, a total of 2240 DEGs were obtained in the cortex I/R group, whereas compared to the striatum sham group, a total of 2595 DEGs were obtained in the striatum I/R group. Hierarchical cluster results showed that the DEGs in the same group preferred to cluster together, but the expression differed at 2, 8, and 24 h post-reperfusion. Thus, these DEGs could distinguish the samples in different groups (Fig. 1).

**Table 2:** The number of differentially expressed genes identified in each group at different ischemia/reperfusion times

	Cortex			Striatum		
	2 h	8 h	24 h	2 h	8 h	24 h
Up	392	509	815	325	538	899
Down	281	401	599	265	524	697
all	673	910	1414	590	1062	1596

### DEGs with a continuous rising and declining trends were identified

**Fig. 2:**
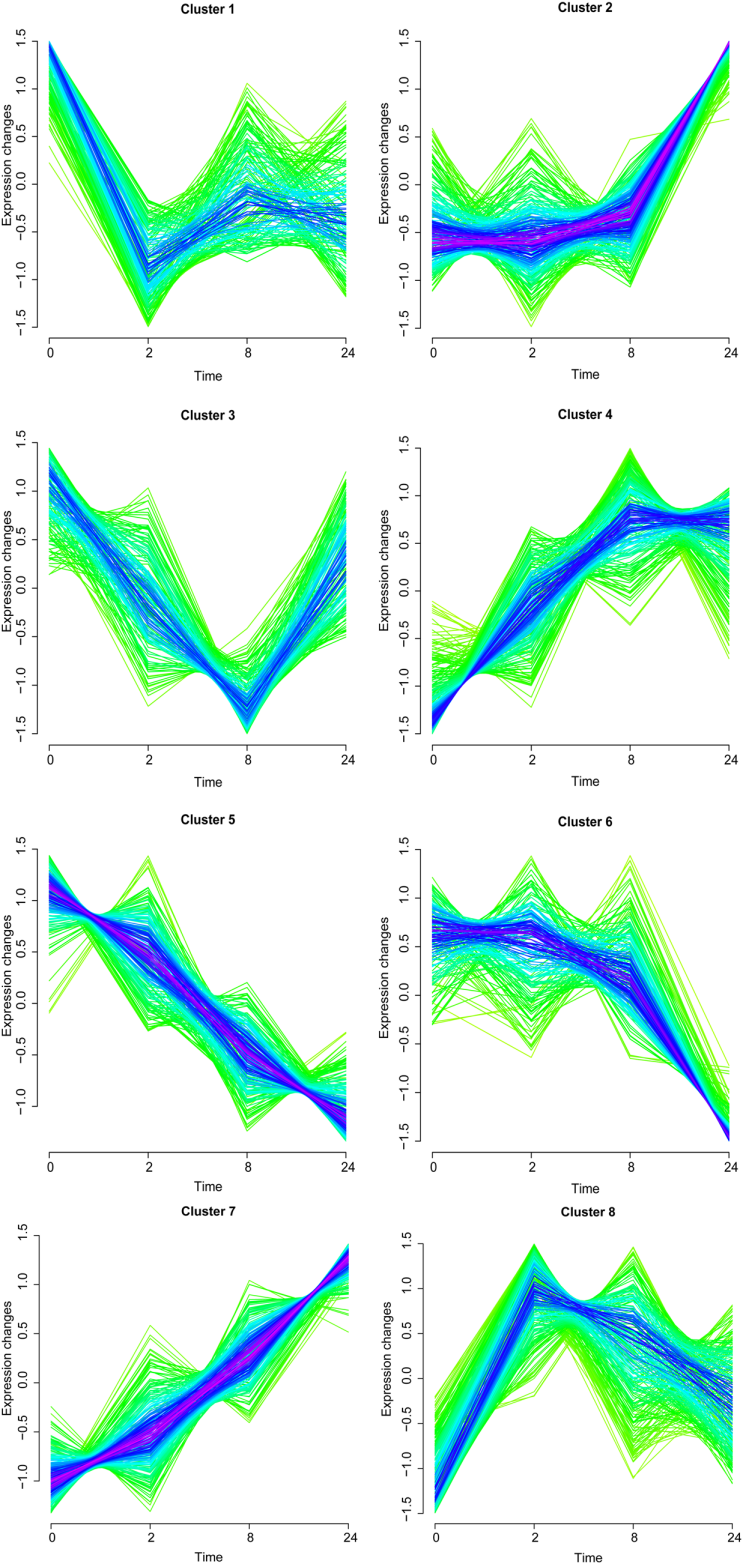
Eight clusters identified in the cortex group based on gene expression changes of DEGs in the cortex at post-reperfusion 0, 2, 8, and 24 h by soft cluster analysis. Yellow or green colored curves correspond to genes with low membership values, while curves in red or purple correspond to genes with high membership values. If genes have a high membership values for a cluster, they are generally similar to each other and contribute significantly to overall expression trends of one cluster. Using this color scheme, clusters with a large core of tightly co-regulated genescan be easily distinguished from week or noisy clusters

**Fig. 3:**
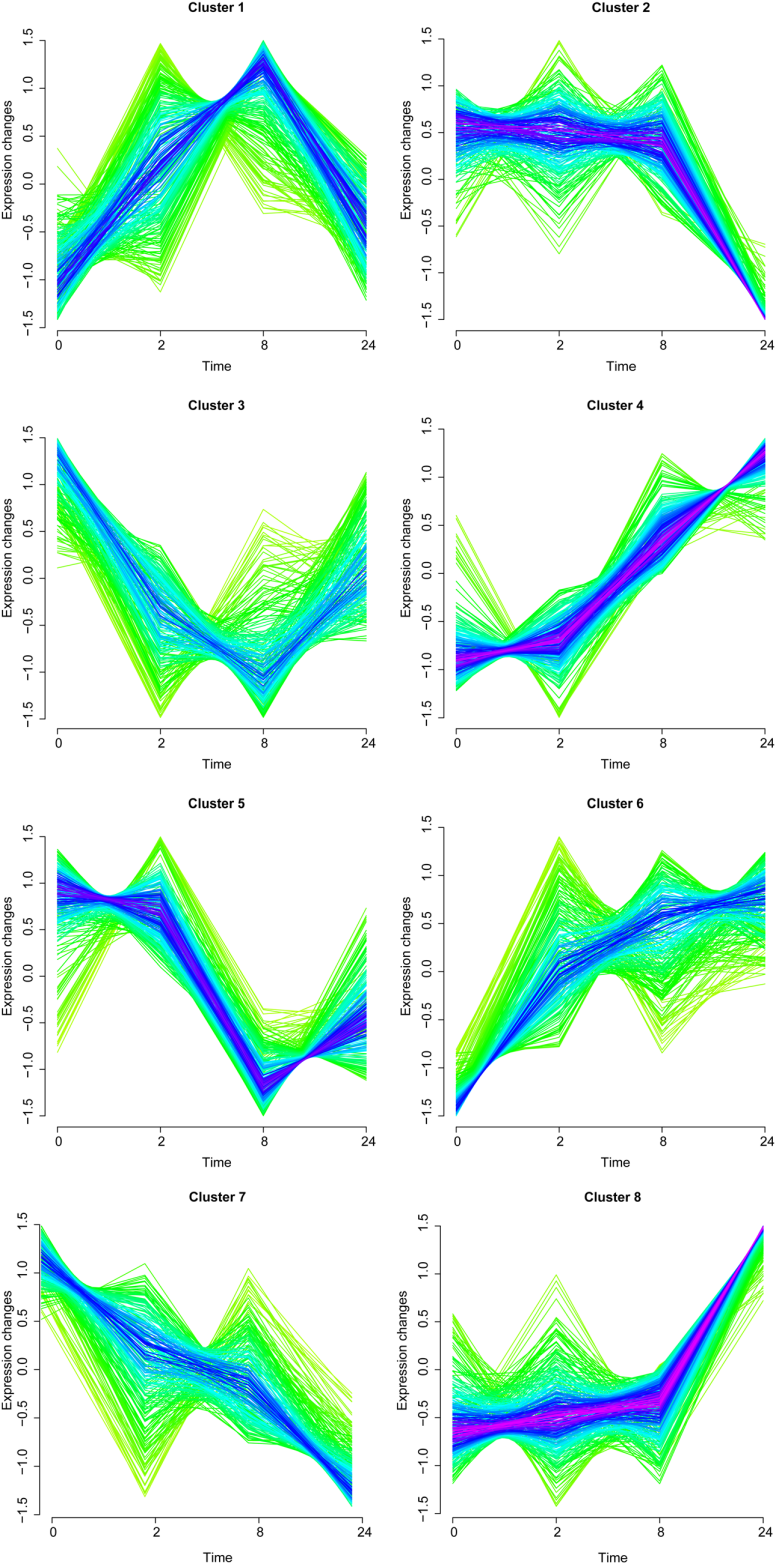
Eight clusters identified in the striatum group based on gene expression changes of DEGs in the striatum at post-reperfusion 0, 2, 8, and 24 h by soft cluster analysis. Curves in yellow or green denote genes with small membership values, while curves in red or purple correspond to genes with big membership values

Based on the time series method in Mfuzz, a total of 8 gene clusters were obtained for the cortex I/R and striatum I/R groups, respectively. With the extension of reperfusion times, different clusters of gene expression changes were identified. In the cortex I/R group, the gene expression values in clusters 2, 4, and 7 showed a rising trend, while those in clusters 5 and 6 showed a downward trend; the gene expression changes in the other clusters were disordered (Fig. 2). In the striatum I/R group, the gene expression values in clusters 4, 6, and 8 showed a rising trend; conversely, clusters 2 and 7 showed a downward trend. In addition, the gene expression values in cluster 1 increased first and then decreased, whereas clusters 3 and 5 decreased first and then increased (Fig. 3). Moreover, in the cortex I/R group, 246 upregulated DEGs were identified in clusters 2, 4, and 7 with a continuous rising trend, and 134 downregulated DEGs in clusters 5 and 6 with a continuous declining trend. In the striatum I/R group, a total of 274 upregulated DEGs were found in clusters 4, 6, and 8 with a continuously rising trend, while 137 downregulated DEGs were found in clusters 2 and 7 with a continuous downward trend (Table 3). Thus, we focused on genes with distinct trends.

**Table 3:** The results of soft clustering analysis for differentially expressed genes identified in each group

	Cortex			Striatum		
	Clusters	Gene counts	Total	Clusters	Clusters	Total
Up	Cluster 2	93	246	Cluster 4	106	274
	Cluster 4	49		Cluster 6	42	
	Cluster 7	104		Cluster 8	126	
Down	Cluster 5	75	134	Cluster 2	98	137
	Cluster 6	59		Cluster 7	39	

### Functional enrichment analysis

The results of GO functional enrichment analysis showed that continuous upregulated DEGs in the cortex group were significantly related to leukocyte migration and response to molecule of bacterial origin, while the continuous downregulated DEGs in cortex group were significantly enriched in the inorganic cation transmembrane transport (*Kcnk3*). In the striatum group, the continuously upregulated DEGs were closely associated with regulation of inflammatory response (C3 and Ctss) angiogenesis (Ecm1 and Hspb6) and response to wounding (Lcp1 and Pros1), but the downregulated DEGs were not significantly enriched in any GO terms (Table 4). For KEGG pathways analysis, the continuous upregulated DEGs in the cortex group were primarily involved in the “TNF signaling pathway (Ccl2, Ccl5, Il6, and Mmp9), whereas the continuously downregulated DEGs in the cortex group were strongly related to cAMP signaling pathway and calcium signaling pathway. In the striatum group, the continuously upregulated DEGs were only enriched in two pathways, including ECM-receptor interaction and proteoglycans in cancer; however, no pathways significantly associated with the downregulated DEGs were detected (Table 5).

**Table 4:** The top five enriched Go terms for continuous up-regulated and down-regulated genes identified in each group listed by p.adjust value

Category	Term	Description	Count	Gene symbol	p.adjust
Up-regulated genes in Cortex group	GO:0,002,237	Response to molecule of bacterial origin	23	Ccl12, Ccl5, Cd14, Cd84, Cebpb, Cxcl16, Il1b, Il6, Lbp, Nos3*…*	1.22E-11
	GO:0,032,496	Response to lipopolysaccharide	22	Nos3, Nr1h3, Nradd, Pycard, Slc11a1, Slpi, Ticam2, Tirap, Tnfrsf1a, Tnfrsf22*…*	1.95E-11
	GO:0,050,900	Leukocyte migration	21	Ecm1, Fcer1g, Fcgr3, Hsd3b7, Icam1, Mmp14, Mmp9, Msn, Pycard, Tirap*…*	4.44E-11
	GO:0,001,819	Positive regulation of cytokine production	23	Bcl3, C3, Ccl2, Ccl5, Cd14, Clec5a, Fcer1g, Tirap, Tlr1, Tnfrsf1a*…*	4.63E-11
	GO:0,002,685	Regulation of leukocyte migration	16	Anxa1, Ccl12, Ccl2, Ecm1, Icam1, Il1b, Lbp, Mmp14, Mmp9,, Tirap*…*	4.37E-10
Down-regulated genes in Cortex group	GO:0,007,611	Learning or memory	12	Drd2, Fgf13, Grin2b, Htr2a, Jph4, Musk, Nf1, Nog, Pak7, Pde1b*…*	7.86E-07
	GO:0,007,613	Memory	9	Cnr1, Cpeb3, Drd2, Fgf13, Grin2b, Htr2a, Musk, Nog, Pak7	8.97E-07
	GO:0,050,890	Cognition	12	Drd2, Fgf13, Grin2b, Htr2a, Jph4, Musk, Nf1, Nog, Pak7, Pde1b*…*	8.97E-07
	GO:0,044,708	Single-organism behavior	13	Alk, Cnr1, Grin2b, Htr2a, Jph4, Musk, Nf1, Nog, Pak7, Pde1b*…*	3.11E-05
	GO:0,098,662	Inorganic cation transmembrane transport	13	Cacng7, Cacng8, Drd2, Htr2a, Kcnd2, Kcnh3, Kcnk3, Pcsk9, Scn4b*…*	3.30E-05
Up-regulated genes in Striatum group	GO:0,031,589	Cell-substrate adhesion	15	Jam3, Msln, Myo1g, Notch1, Otoa, Plau, S100a10, Sdc4, Spp1, Tnn*…*	2.69E-05
	GO:0,007,160	Cell–matrix adhesion	11	Col3a1, Gpm6b, Iqgap1, Jam3, Msln, Otoa, Plau, S100a10, Sdc4, Tnn*…*	0.000111
	GO:0,009,611	Response to wounding	16	Hspb1, Lcp1, Lox, Map2k1, Plau, Pros1, S100a9, Sdc4, Slc11a1, Tnc*…*	0.000111
	GO:0,050,727	Regulation of inflammatory response	13	C3, Casp1, Ctss, Fcgr2b, Mefv, Myd88, Nr1d2, S100a8, S100a9, Tlr3*…*	0.000111
	GO:0,001,525	Angiogenesis	16	Esm1, Hmox1, Hspb1, Hspb6, Ihh, Jam3, Lif, Lrg1, Notch1, Thbs2*…*	0.000111

Note: *GO* Gene ontology

**Table 5:** The top five enriched pathways for continuous up-regulated and down-regulated genes identified in each group listed by p.adjust value

Category	Term	Description	Count	Gene symbol	p.adjust
Up-regulated genes in Cortex group	mmu04668	TNF signaling pathway	12	Bcl3, Ccl12, Ccl2, Ccl5, Cebpb, Icam1, Il1b, Il6, Mlkl, Mmp14, Mmp9, Tnfrsf1a	1.07E-05
	mmu05144	Malaria	8	Ccl12, Ccl2, Hba-a1, Hbb-b1, Icam1, Il1b, Il6, Selp	4.80E-05
	mmu05133	Pertussis	9	C1qb, C3, C4b, Cd14, Il1b, Il6, Pycard, Ticam2, Tirap	9.08E-05
	mmu05152	Tuberculosis	13	C3, Cd14, Cebpb, Fcer1g, Fcgr1, Fcgr3, Il1b, Il6, Lbp, Lsp1, Tirap, Tlr1, Tnfrsf1	0.000101
	mmu05150	Staphylococcus aureus infection	7	C1qb, C3, C4b, Fcgr1, Fcgr3, Icam1, Selp	0.000337
Down-regulated genes in Cortex group	mmu04024	cAMP signaling pathway	8	Atp2b1, Atp2b4, Drd2, Gria3, Grin2b, Htr1d, Rock2, Sstr2	0.00716
	mmu05033	Nicotine addiction	4	Gabrg1, Gria3, Grin2b, Slc17a8	0.009442
	mmu04020	Calcium signaling pathway	7	Atp2b1, Atp2b4, Htr2a, Mylk2, Pde1b, Plcz1, Ryr1	0.010304
	mmu05032	Morphine addiction	5	Gabrg1, Gnb4, Pde10a, Pde1b, Pde7b	0.012376
	mmu04261	Adrenergic signaling in cardiomyocytes	6	Atp2b1, Atp2b4, Cacna2d1, Cacng7, Cacng8, Scn4b	0.012376
Up-regulated genes in Striatum group	mmu04512	ECM-receptor interaction	6	Col1a1, Sdc4, Spp1, Thbs2, Tnc, Tnn	0.025936
	mmu05205	Proteoglycans in cancer	9	Col1a1, Ihh, Iqgap1, Map2k1, Msn, Plau, Plaur, Rras2, Sdc4	0.025936

### Identification of key DEGs

**Fig. 4:**
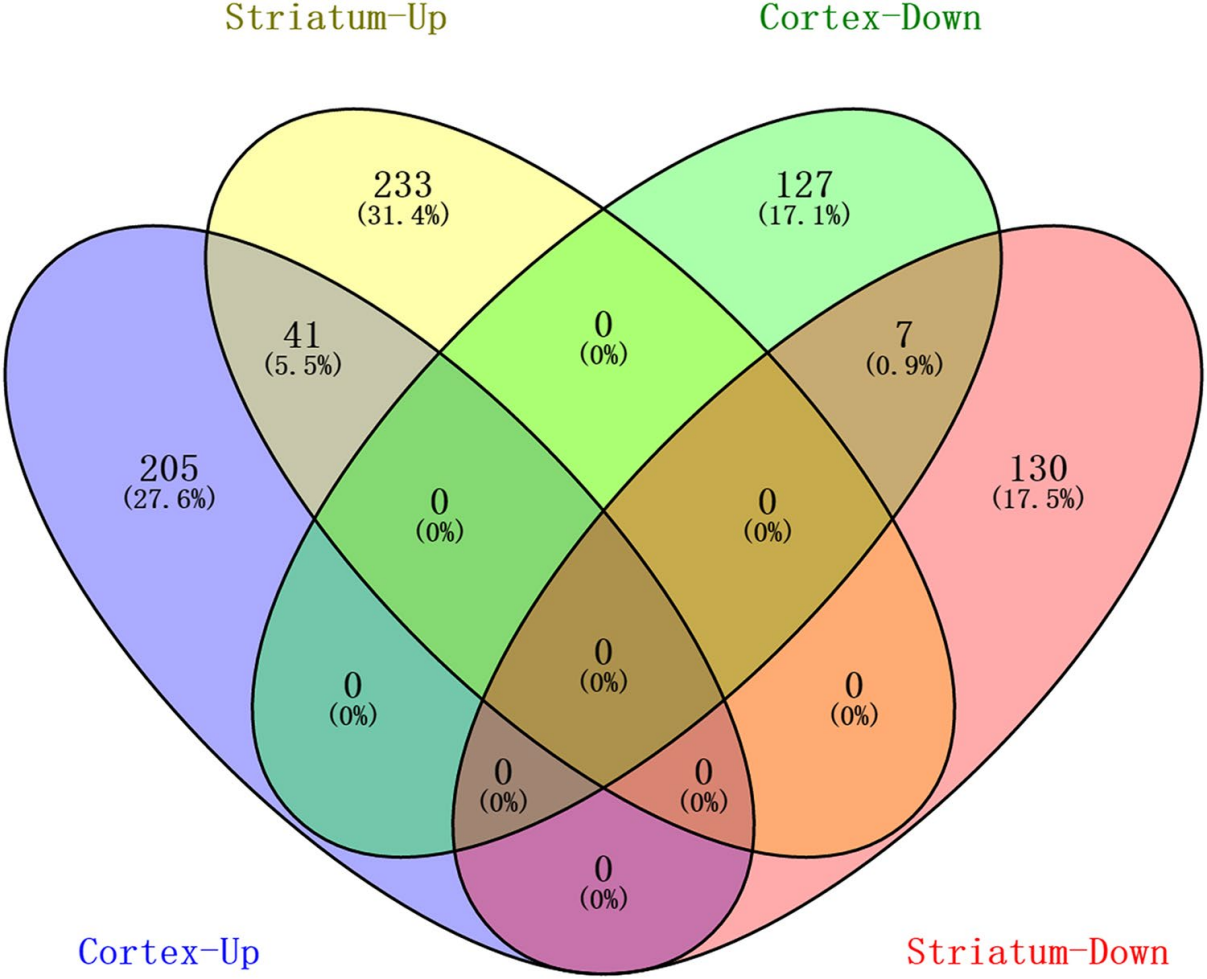
The Venn diagram of overlapped upregulated genes and shared downregulated genes in both cortex and striatum groups, respectively. A total of 41 overlapped upregulated DEGs and 7 overlapped downregulated DEGs were identified in both groups

**Fig. 5:**
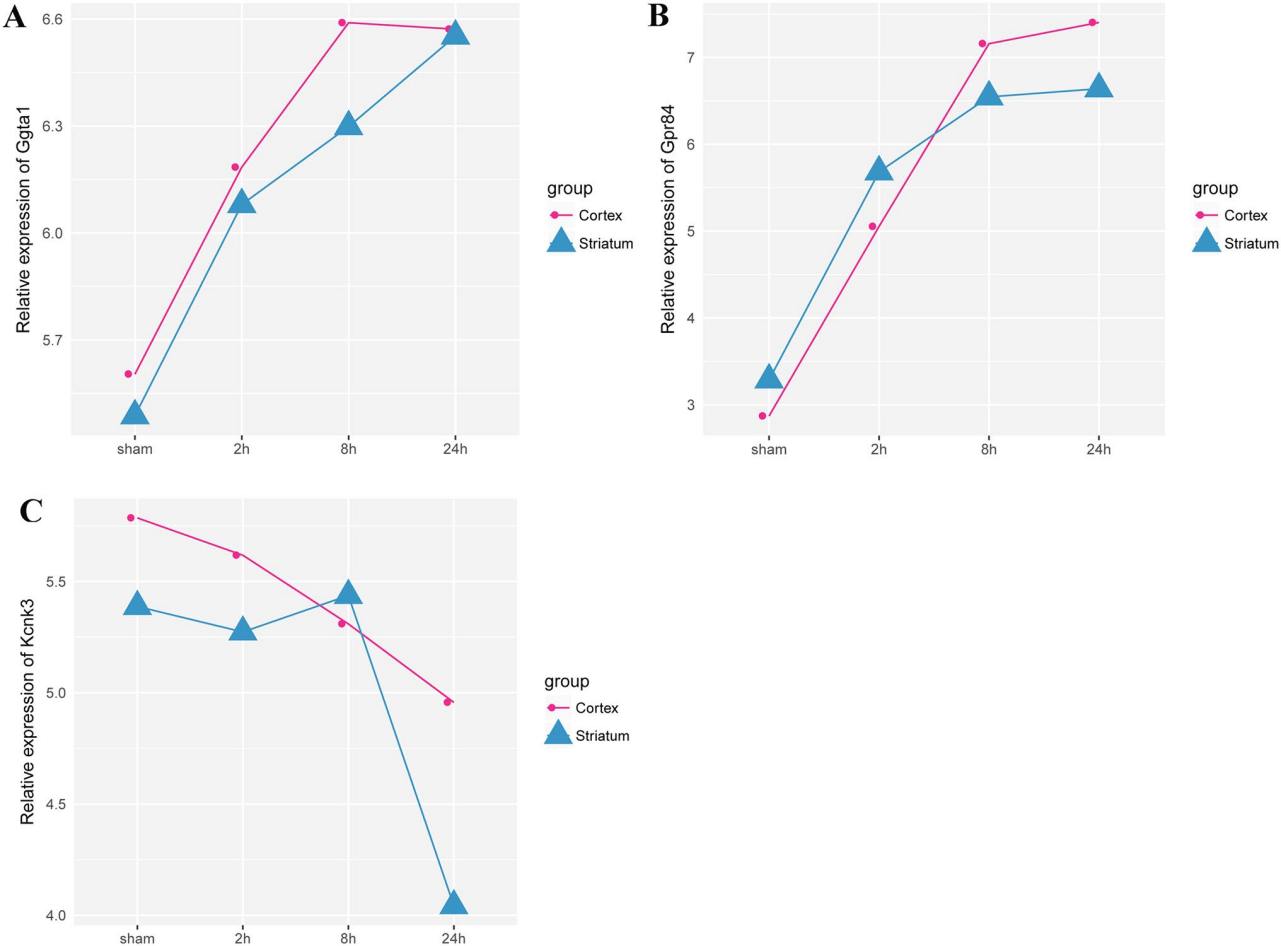
The expression trends of *Ggta1* (**A**), *Gpr84* (**B**), and *Kcnk3* (**C**) at 2, 8, and 24 h post-reperfusion identified in both cortex and striatum groups. The expression of *Ggta1* and *Gpr84* was upregulated with prolonged post-reperfusion time, while *Kcnk3* presented downregulation prolonged post-reperfusion time except for post-reperfusion 8 h in the striatum group

To explore the common change in genes with continuous rise and fall in both cortex and striatum groups, a Venn diagram analysis was used. A total of 41 overlapped upregulated DEGs and 7 overlapped downregulated DEGs were found (Fig. 4). The expression of 5/41 DEGs (*Timp1*, *Gpr84*, *Ggta1*, *4930486L24Rik*, and *Osmr*) was predicted to have a significant difference at each reperfusion time point in the cortex and striatum groups as compared to the sham group. Moreover, the expression trend of *Ggta1* and *Gpr84* was significantly lower P-value at each reperfusion time point in the cortex and striatum groups as compared to the sham group (Fig. 5A and B). However, none of the downregulated DEGs exhibited significant difference at each reperfusion time in cortex while comparing the striatum to the sham group, and the expression of *Kcnk3* was downregulated except post-reperfusion at 8 h in the striatum group (Fig. 5C).

### Validation by real-time PCR and western blotting

**Fig. 6:**
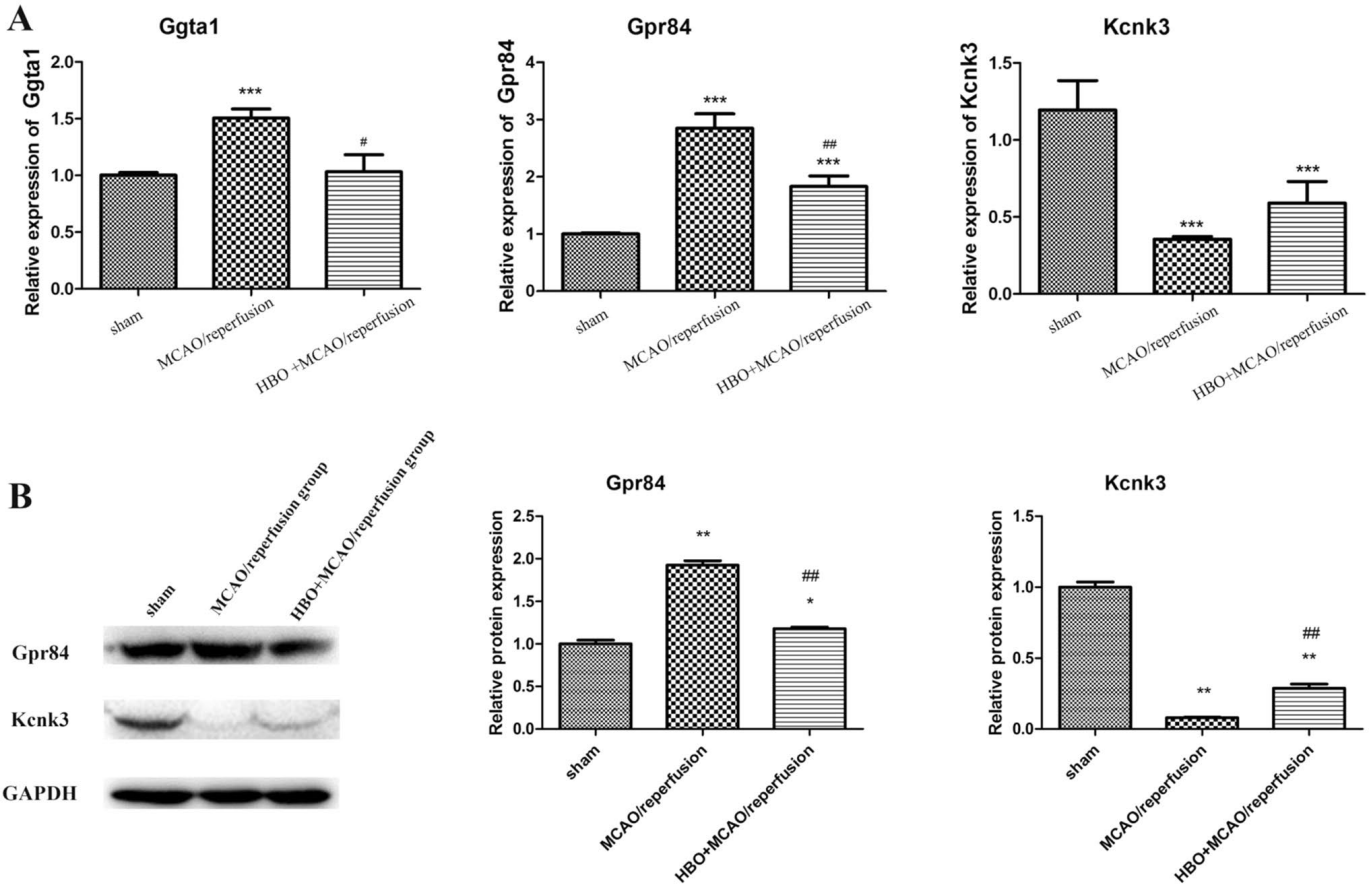
The relative expression of key genes. The mRNA levels of *Ggta1*, *Gpr84*, and *Kcnk3* (**A**) detected by real-time PCR. The protein levels of *Gpr84* and *Kcnk3* measured by western blotting. *MCAO* middle cerebral artery occlusion, *HBO* hyperbaric oxygenation. *, **, and *** represent *P* < 0.05, *P* < 0.01, and *P* < 0.001 *vs*. sham operation group, respectively. #, ##, and ### represent *P* < 0.05, *P* < 0.01, and *P* < 0.001 *vs*. MCAO/reperfusion group, respectively

The expression of three genes (*Ggta1*, *Gpr84*, and *Kcnk3*), was distinct as evaluated by RT-PCR in MCAO/reperfusion, sham operation, and HBO preconditioning + MCAO/reperfusion groups (Fig. 6A). The results showed that *Ggta1* mRNA level was significantly upregulated in MCAO/reperfusion rats (*P* < 0.001 *vs*. sham), and HBO preconditioning markedly attenuated the *Ggta1* expression in MCAO/reperfusion rats (*P* < 0.05). Similarly, a significantly increased mRNA expression of *Gpr84* was detected in MCAO/reperfusion rats (*P* < 0.001 *vs*. sham), and HBO preconditioning suppressed the expression of *Gpr84* in MCAO/reperfusion rats significantly (*P* < 0.01). In addition, the mRNA expression of *Kcnk3* was significantly downregulated in MCAO/reperfusion rats (*P* < 0.001 *vs*. sham), and after HBO preconditioning, the expression was only slightly upregulated in these rats.

In keeping with the RT-PCR analysis, the data of western blotting revealed that the protein level of Gpr84 in MCAO/reperfusion rats was significantly upregulated in comparison to that in sham controls (*P* < 0.01; Fig. 6B); moreover, HBO preconditioning could significantly suppress the expression of Gpr84 protein in MCAO/reperfusion rats (P < 0.01). In contrast, the expression of Kcnk3 protein in MCAO/ reperfusion rats was significantly down-regulated (*P* < 0.01 *vs*. sham), while it was up-regulated after HBO pretreatment (*P* < 0.01).

## Discussion

In the present study, a set of 41 overlapped upregulated DEGs and 7 overlapped downregulated DEGs was found in the cortex and striatum groups, among which the upregulated expression of *Gpr84* and *Ggta1* was significantly different at each reperfusion time points in both groups, while that of downregulated *Kcnk3* was distinct. RT-PCR and western blotting analyses showed that the expression of the *Ggta1*, *Gpr84*, and *Kcnk3* genes between MCAO/reperfusion and sham rats was consistent with the results of bioinformatics analysis described above. In addition, the expression of these three genes was significantly altered after HBO preconditioning. The current findings revealed new genes that might play critical roles in CIRI, and the protective effect induced by HBO preconditioning might be associated with DEGs such as *Ggta1*, *Gpr84*, and *Kcnk3* for ameliorating the subsequent CIRI.

Glycoprotein, alpha-*galactosyltransferase* 1 pseudogene (*Ggta1*, also named *GLYT2*) encodes a terminal glycosyltransferase, which generates a carbohydrate antigen; namely, α-gal epitopes (Galα1-3Galβl-4GlcNAc-R) (Shaper et al. 1992). Typically, *Ggta1* is inactivated in human beings, which might lead to remove the α-gal epitopes and produce abundant natural anti-Gal antibody in the human (Galili 2015). Conversely, the activated *Ggta1* may reduce the abundance of natural anti-Gal antibody. anti-Gal antibody is shown to maintain the immunological balance between autoimmunity and infection in humans (Jaison et al. 1993), and *Ggta1* knockout in mice promotes anti-Gal-mediated increase in immunogenicity (Abdelmotal et al. 2009). In addition, *Ggta1* is reported to be overexpressed after traumatic brain injury in rats (Samal et al. 2015). In the present study, we detected that the expression of *Ggta1* was significantly upregulated in the MCAO/reperfusion group, and the expression was significantly reduced after HBO preconditioning. Thus, we speculated that the downregulated *Ggta1* induced by HBO preconditioning might ameliorate the brain damage in MCAO/reperfusion rats with decreased immunogenicity. However, additional experiments are required to verify this hypothesis.

G-protein-coupled receptor 84 (*Gpr84*) is a receptor of medium-chain free fatty acid with a crucial role in fatty acid metabolism that regulates the immune system (Suzuki et al. 2013). Masakatsu et al. reported that the activated *Gpr84* as a proinflammatory receptor and may not only accumulate the polymorphonuclear leukocytes and macrophages but also mediate lipopolysaccharide-induced IL-8 and TNF-α production (Suzuki et al. 2013). In addition, Venkataraman et al. demonstrated that in GPR84-knockout T cells, the expression of anti-inflammatory factor IL-4 is increased, which implied that the overexpression of *GPR84* mediates the downregulation of IL-4 and in turn, promotes inflammatory response (Venkataraman and Kuo 2005). Inflammation is the main pathogenesis for cerebral ischemia, and a marked inflammatory reaction is evoked by cerebral I/R with increased pro-inflammatory factors, such as TNFα, IL-1β, and IL-6 (Collino et al. 2006; Junjie et al. 2016). Moreover, the IL-4 knockout after cerebral ischemia might increase brain damage and inflammation (Xiong et al. 2011). In the present study, *Gpr84* was found to be significantly upregulated in MCAO/reperfusion rats as compared to sham rats; however, after HBO preconditioning, the expression of *Gpr84* was decreased. Thus, we speculated that the HBO preconditioning-induced decrease in *Gpr84* might protect the brain against CIRI by reducing the expressions of inflammation molecules. However, additional experiments are needed to verify this hypothesis.

Potassium channel subfamily K member 3 (*KCNK3*, also known as *TASK-1*) belongs to a member of potassium channel proteins, and the suppression of *KCNK3* acts as a leading player in the pathogenesis of pulmonary arterial hypertension pathogenesis with increased proliferation and inflammation (Antigny et al. 2016). Muhammad et al. found that *KCNK3* knockout in rat brains might expand the infarct volumes, resulting in a large brain injury, thereby influencing the blood vessel microarchitecture and blood flow rate (Muhammad et al. 2010). Consistent with the current findings, *KCNK3* was significantly downregulated in MCAO/reperfusion rats, and HBO preconditioning increased the expression of *KCNK3* in MCAO/reperfusion rats slightly. Several studies suggested that *KCNK3* is a key form of oxygen-sensitive background K + channel in rat carotid body glomus cells, and hypoxia can induce the inhibition of *TASK-1*-like channels (Kim et al. 2009; PJ and KJ 2013). Collectively, we inferred that the upregulation of *KCNK3* induced by HBO preconditioning ameliorates the brain damage in MCAO/reperfusion rats. However, the underlying mechanism needs to be elucidated further.

In summary, the current study showed that HBO treatment might reduce brain damages and protect the brain against CIRI via altered gene expression alteration of *Ggta1*, *Gpr84*, and *Kcnk3*. Nonetheless, the protective mechanisms and pathways of *Ggta1*, *Gpr84*, and *Kcnk* against CIRI after HBO treatment need further investigation.

## Data Availability

The datasets used and/or analysed during the current study are available from the corresponding author on reasonable request.
